# Predictive factors of successful endovascular recanalization for symptomatic atherosclerotic non-acute middle cerebral artery occlusion

**DOI:** 10.3389/fneur.2025.1650169

**Published:** 2025-09-11

**Authors:** Lin Ma, Hao Zhang, Shuo Yan, Weixing Bai, Yingqiang Zhang, Dedi Wu, Jiahang Du, Zhiyong Lu, Huaqiao Tan

**Affiliations:** ^1^Department of Interventional Radiology, The Seventh Affiliated Hospital, Sun Yat-sen University, Shenzhen, China; ^2^Department of Interventional Radiology, Tongji Hospital, School of Medicine, Tongji University, Shanghai, China

**Keywords:** endovascular recanalization, middle cerebral artery, atherosclerosis, non-acute occlusion, predictors

## Abstract

**Background:**

Endovascular recanalization treatment (EVRT) has emerged as a critical treatment modality for patients with symptomatic atherosclerotic non-acute middle cerebral artery occlusion (SNMCAO). However, several technical challenges persist, and the predictive factors for successful recanalization of SNMCAO are not yet fully understood. This study aimed to identify clinical and radiological factors associated with successful EVRT in patients with SNMCAO.

**Methods:**

We conducted a retrospective analysis on all patients with SNMCAO who underwent EVRT at two centers from January 2016 to December 2024. Demographic data, medical history, imaging characteristics, periprocedural complications, and 3-month follow-up results were collected. Logistic binary regression analysis was performed to assess the factors influencing the success of EVRT.

**Results:**

A total of 65 patients were included, with 50 achieving successful recanalization. The perioperative complication rate was 13.8% (9/65). Multivariate logistic binary regression analysis indicated that an occlusion duration of ≤ 3 months (*P* = 0.020), an occlusion segment length of ≤ 10 mm (*P* = 0.004), the presence of a distal vascular main trunk visualization (DVMTV) sign (*P* = 0.035), and the presence of a slow distal antegrade flow (SDFA) sign (*P* = 0.039) were identified as independent positive predictors of successful EVRT for SNMCAO.

**Conclusion:**

Occlusion duration of ≤ 3 months, occlusion length of ≤ 10 mm, the presence of a DVMTV sign and a SDFA sign were considered independent predictive factors for the success of EVRT in patients with SNMCAO.

## Introduction

Although the time window for endovascular recanalization treatment (EVRT) of acute intracranial large artery occlusion has been extended to 24 h post-onset after screening, in reality, the proportion of patients who can reach the hospital within this timeframe and receive active treatment remains very low, at < 5% in China. Cases in which intracranial large artery occlusion is detected 24 h after the onset of symptoms are typically classified as non-acute occlusion cases. A study utilizing neuroimaging indicates that the incidence of intracranial artery large occlusion in Asian stroke patients is as high as 34.5% (1). According to the Chinese IntraCranial AtheroSclerosis Study and other studies, the incidence of middle cerebral artery (MCA) occlusion is higher than that of other intracranial arteries, with atherosclerosis being the most common cause (2). Patients with non-acute MCA occlusion, especially those with compromised hemodynamics or inadequate collateral circulation compensation, continue to face a high risk of stroke recurrence despite optimal medical treatment (2–4). Furthermore, the RECON trial confirmed that long-term cerebral hypoperfusion leads to cognitive and emotional disorders in patients with intracranial large artery occlusion (5). However, there is currently no consensus on the optimal treatment for symptomatic non-acute MCA occlusion (SNMCAO). In recent years, several clinical studies have reported that EVRT for SNMCAO has demonstrated a certain level of safety and effectiveness, which can improve patients' ischemic symptoms and reduce the recurrence rate of stroke (6–10). Due to the distinct pathophysiology of non-acute occlusion compared to acute occlusion, EVRT for SNMCAO is technically challenging and carries risks, potentially leading to life-threatening complications. Therefore, selecting the appropriate subgroup of SNMCAO patients who can benefit from EVRT is key to reducing surgical risks and improving the technical success rate. In this study, we conducted a systematic retrospective analysis of SNMCAO patients who underwent EVRT at two centers, aiming to identify predictive factors for successful endovascular recanalization in these patients, thereby providing a reference for selecting SNMCAO patients suitable for EVRT.

## Methods

### Patient selection and management

**Figure 1:**
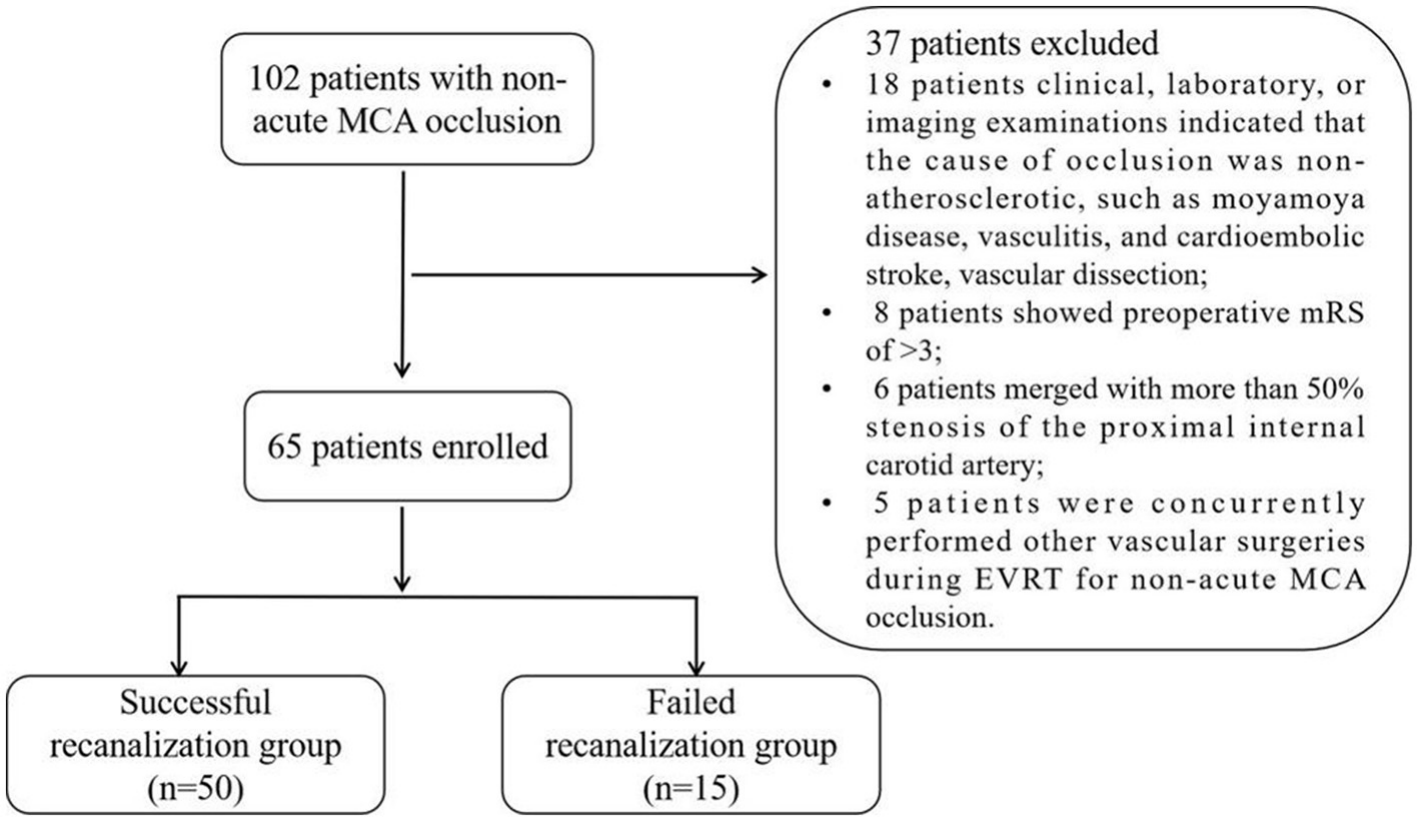
Flow chart of patient enrollment.

This study conducted a retrospective analysis on all patients with SNMCAO who underwent EVRT consecutively from January 2016 to December 2024 at the Seventh Affiliated Hospital of Sun Yat-sen University and the Tongji Hospital Affiliated to Tongji University. A flowchart of the study is provided in Figure 1. This study received approval from the institutional review board, and informed consent was waived due to the retrospective nature of the analysis.

The inclusion criteria for SNMCAO patients undergoing EVRT were as follows: (1) A stroke or transient ischemic attack (TIA) lasting over 24 h, diagnosed with an occlusion of the M1 segment of the MCA via computed tomography angiography (CTA), and confirmed by digital subtraction angiography (DSA); (2) At least one atherosclerotic risk factor; (3) Stroke or TIA related to the side of the MCA occlusion still occurred after aggressive medical treatment including dual antiplatelet, lipid-lowering, and other risk factor management; (4) Cerebral perfusion examination (CTP) shows low perfusion in the occluded MCA area [prolonged time to peak (TTP) and mean transit time (MTT), decreased cerebral blood flow (CBF), and normal or decreased cerebral blood volume (CBV)]; (5) A preoperative Modified Rankin Score (mRS) of ≤ 3. The exclusion criteria were established to meet the following conditions: (1) Clinical, laboratory, or imaging examinations indicated that the cause of occlusion was non-atherosclerotic, such as moyamoya disease, vasculitis, and cardioembolic stroke, etc.; (2) Merged with more than 50% stenosis of the proximal internal carotid artery; (3) The angle of occlusion segment was more than 90°; (4) Severe calcification defined by an arc of calcification >180° at the occluded segment; (5) Merged with more than 50% stenosis of the proximal internal carotid artery; (6) During EVRT for non-acute MCA occlusion, other vascular surgeries were concurrently performed; (7) The patient's life expectancy was < 1 years.

The specific details of the patient's preoperative preparation, EVRT procedure, and postoperative management were available in our previous research (11, 12).

### Definition of variables related to EVRT

**Figure 2:**
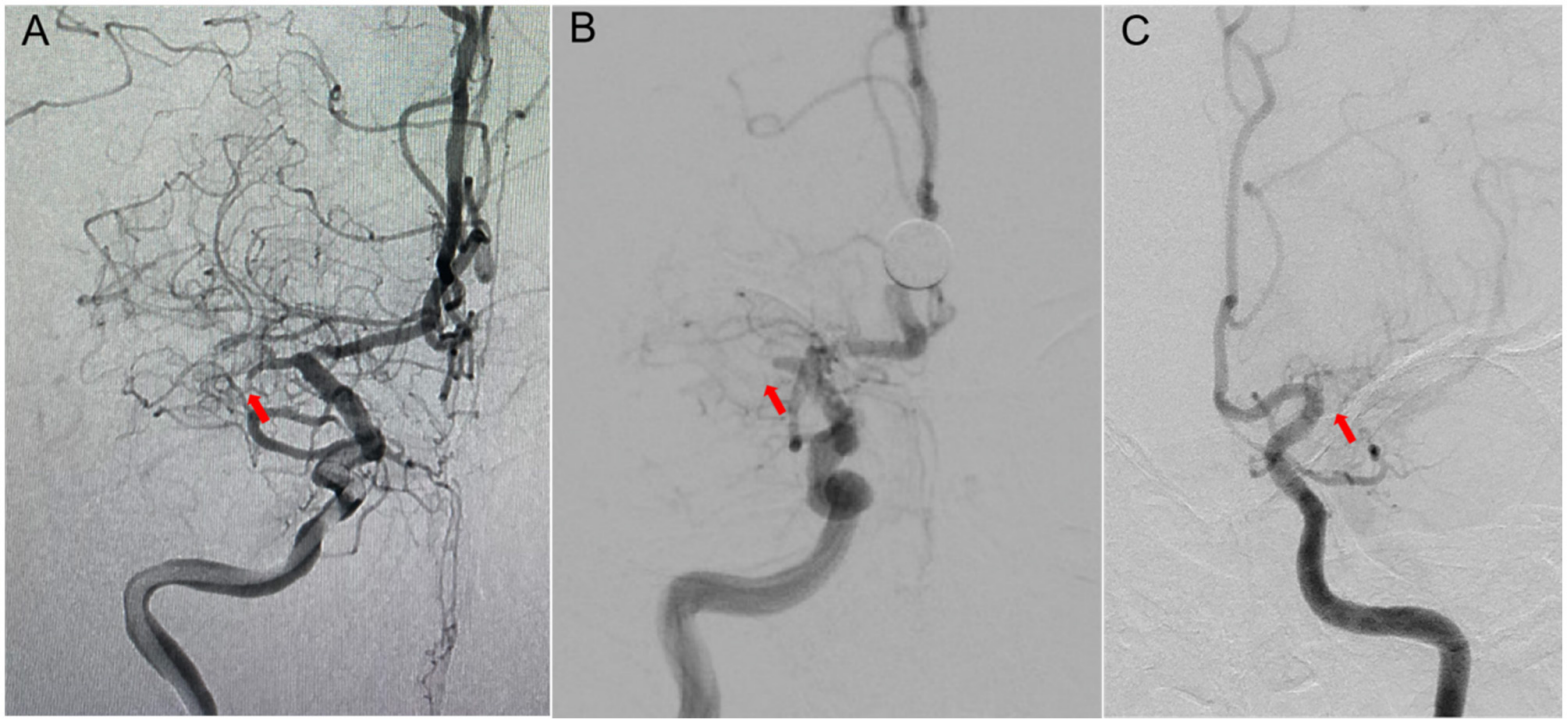
The different stump morphology of the occluded segment. **(A)** Right internal carotid artery (ICA) angiography showed occlusion of M1 segment of the right middle cerebral artery (MCA), with a tapered-stump of the occluded segment (red arrow). **(B)** Right ICA angiography showed occlusion of M1 segment of the right MCA, with a blunt-stump of the occluded segment (red arrow). **(C)** Left ICA angiography showed occlusion of M1 segment of the left MCA, with no stump of the occluded segment (red arrow).

**Figure 3:**
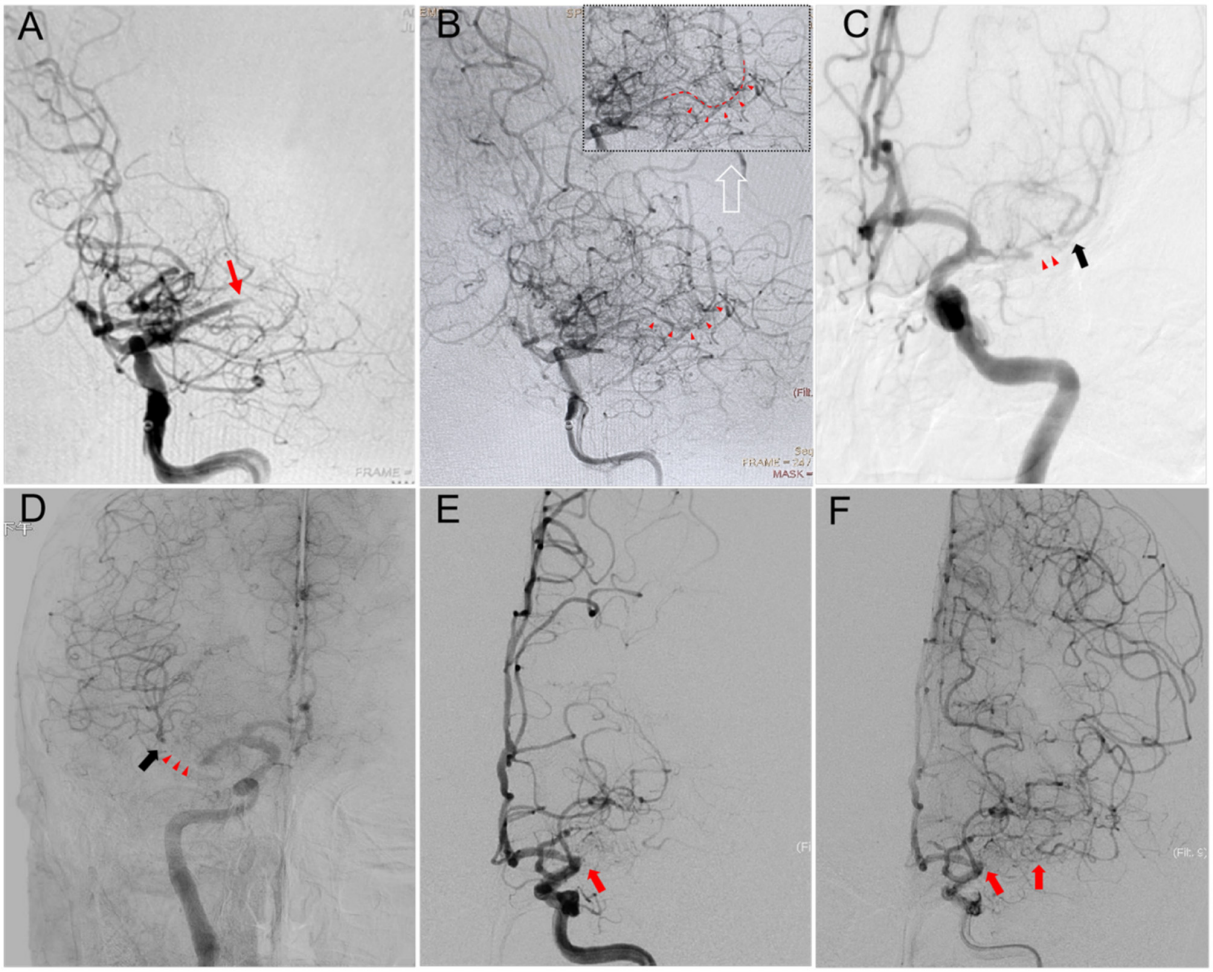
**(A)** Left internal carotid artery (ICA) angiography of a successful recanalization case showed occlusion of M1 segment of the left middle cerebral artery (MCA), with a tapered-stump of the occluded segment (red arrow). **(B)** A slow antegrade flow (SDFA sign) through the occluded segment, with visualization of the filling of the distal M2 segment vessels (indicated by red arrows). **(C)** Left ICA angiography of a successful recanalization case showed occlusion of M1 segment of the left MCA, and a distal vascular main trunk visualization (DVMTV) sign (black arrow) at the mid-arterial phase images through collateral circulation, without a SDFA sign (red arrow). **(D)** Right ICA angiography of a successful recanalization case showed occlusion of M1 segment of the right MCA, and a DVMTV sign (black arrow) at the late arterial phase images through retrograde blood flow from anterior cerebral artery, but without a SDFA sign (red arrow). **(E, F)** The right ICA angiography of a failed recanalization case revealed occlusion of the M1 segment of the right MCA, with no stump present and lacking the SDFA sign and the DVMTV sign (red arrow).

Successful recanalization was defined as a residual stenosis of ≤ 30%, and an antegrade blood flow with a modified Thrombolysis in Cerebral Infarction (mTICI) score of ≥2b (13). The duration of MCA occlusion in patients was estimated based on the onset of clinical events or determined by the results of the patient's previous imaging examinations, and was classified as ≤ 3 and >3 months. The morphology of the MCA occlusion stump was assessed using DSA angiography. If the occlusion stump exhibited a funnel-shaped morphology, it was classified as a “tapered” stump; otherwise, it was classified as a “blunt” stump or no stump (Figure 2). This classification was based on the morphology of the occluded stump in cases of coronary artery chronic total occlusions (CTO) (14). The length of the occluded segment was defined as the absence of contrast agent filling on the multi-planar reconstruction images of cerebral CTA, and it was classified according to the length of the total cavity filling defect as ≤ 10 mm or >10 mm. The slow distal antegrade flow (SDAF) sign referred to the slow antegrade flow of contrast agent at the distal end of the occlusion site during the late arterial phase of angiography (Figure 3). It was categorized into two groups: the absence of the SDAF sign and the presence of the SDAF. The distal vascular main trunk visualization (DVMTV) sign referred to the imaging of the distal M1 main trunk vessel or the superior and inferior trunk vessels by the slow antegrade flow or retrograde collateral circulation filling of the contrast agent (Figure 3). It was categorized into the absence of the DVMTV group and the presence of the DVMTV group.

### Data collection and statistical analysis

The case data for this study were sourced from the endovascular interventional treatment databases of two centers specializing in intracranial artery occlusion. The demographic data, clinical baseline characteristics, preoperative imaging features, endovascular treatment outcomes, and perioperative complications of the research subjects were meticulously recorded. The preoperative imaging characteristics included the morphology of the occlusion stump, the length of the occluded segment, the DVMTV sign and the SDAF sign. The outcomes of interventional treatment included the method of recanalization, the success of recanalization, and the postoperative perfusion status. Follow-up data were collected and recorded based on subsequent clinical manifestations and angiographic results. The assessment of all imaging examination data was completed by two independent, senior neurointerventional radiology experts, and any differences were resolved through their final consensus.

The statistical analysis was conducted using SPSS Statistics 27.0 software. Categorical variables were presented as frequencies or percentages. Continuous numerical variables that adhered to a normal distribution were described using the mean ± standard deviation (*x* ± *s*), whereas those that did not conform to a normal distribution were described using the median and interquartile range. The independent samples *t*-test or Mann–Whitney *U*-test was used to compare the characteristics of continuous numerical variables across different groups. The Pearson Chi-square test or Fisher's exact test was used to analyze the characteristics of categorical variables across different groups. Logistic binary regression analysis was used to investigate factors influencing successful endovascular recanalization. In the multivariate model, only factors with a *p*-value < 0.05 from the univariate model were included for further analysis. The outcomes of the regression analysis were presented using the odds ratio (OR) and the corresponding 95% confidence interval (CI), along with the *p*-value. A *p*-value < 0.05 was considered statistically significant.

## Results

### Baseline characteristics

A total of 102 patients' clinical and imaging data were obtained. After analysis, 65 patients met the inclusion criteria and were included in this study. The clinical baseline characteristics of the patients are detailed in Table 1. Of these cases, 47 (72.3%) involved male patients, aged between 38 and 78 years (median age, 62 years). Twenty-eight patients experienced recurrent TIA attacks or stroke progression during their hospital stay. Additionally, 37 patients were readmitted due to a recurrent stroke or TIA. 70.8% (46/65) of the patients had hypertension, 40.0% (26/65) had diabetes, 49.2% (32/65) had hyperlipidemia, 27.7% (18/65) had coronary heart disease, and 50.8% (33/65) had a history of smoking. The median time from the discovery of MCA occlusion to the initiation of EVRT was 46 days [interquartile range (IQR), 16–72 days), with occlusion times of ≤ 3 months in 52 patients, and >3 months in 13 patients. The median occlusion length was 7.6 mm (ranging from 3.5 to 15.5 mm), with occlusion lengths of ≤ 10 mm in 41 patients, and >10 mm in 24 patients. Of the patients, 58.5% (38/65) had a tapered stump, 50.8% (33/65) exhibited a DVMTV sign, and 36.9% (24/65) showed a SDAF sign at the distal end of the occlusion site (Table 1).

**Table 1:** Baseline characteristics of patients with SNMCAO.

**Variables**	**Total (*n* = 65)**	**Successful group (*n* = 50)**	**Failed group (*n* = 15)**	***P* value**
Age, mean (SD), y	60.2 ± 9.4	59.8 ± 9.4	61.7 ± 9.5	0.485
Male, *n* (%)	47 (72.3)	37 (74.0)	10 (66.7)	0.579
**Risk factors**, ***n*** **(%)**				
Hypertension	46 (70.8)	36 (72.0)	10 (66.7)	0.691
Diabetes mellitus	26 (40.0)	21 (42.0)	5 (33.3)	0.549
Dyslipidemia	32 (49.2)	26 (52.0)	6 (40.0)	0.417
Coronary heart disease	18 (27.7)	14 (28.0)	4 (26.7)	0.919
Smoking history	33 (50.8)	26 (52.0)	7 (46.7)	0.717
**Qualifying event**, ***n*** **(%)**				
Progressive stroke	23 (35.4)	18 (36.0)	5 (33.3)	0.850
Recurrent stroke/TIA	42 (64.6)	32 (64.0)	10 (66.7)	
Estimated occlusion duration (≤3 months), *n* (%)	52 (80.0)	43 (86.0)	9 (60.0)	0.034
Occlusion length (≤10 mm), *n* (%)	41 (63.1)	35 (70.0)	6 (40.0)	0.040
Tapered-stump, *n* (%)	38 (58.5)	33 (66.0)	5 (33.3)	0.030
DVMTV sign, *n* (%)	33 (50.8)	29 (58.0)	4 (26.7)	0.040
SDAF sign, *n* (%)	24 (36.9)	22 (44.0)	2 (13.3)	0.044
Preoperative NIHSS score, median (IQR)	4 (2-7)	4 (2–6)	4 (2–8)	0.996
Preoperative mRS score, median (IQR)	2 (1-2.5)	2 (1–2)	2 (1–3)	0.797

SNMCAO, symptomatic non-acute middle cerebral artery occlusion; IQR, interquartile range; SD, standard deviation; TIA, transient ischemic attack; SDAF, slow distal antegrade flow; DVMTV, distal vascular main trunk visualization.

### Primary procedural outcomes and perioperative complications

Out of the 65 total cases, 50 were successfully recanalized, while 15 cases failed. Of the 50 patients who experienced successful recanalization, 10 underwent simple balloon dilation angioplasty, 25 received balloon-expandable stents subsequent to balloon dilation, and 15 had balloon dilation followed by the implantation of self-expanding stents. Among the 15 patients with failed recanalization, 10 cases were terminated due to repeated unsuccessful attempts, 2 cases were terminated due to microguidewire-induced vessel perforation, one case underwent MCA embolization treatment due to a lenticulostriate artery hemorrhage after recanalization, and 2 cases had an mTICI grade of 2a after recanalization.

A total of 9 procedural complications occurred, with a complication rate of 13.8%. Among these, 6 cases occurred in the successful EVRT group, while 3 cases were observed in the failed group. However, only 2 cases (3.1%) were discharged with symptomatic complications. Among the 50 patients who underwent successful EVRT, two developed hyperperfusion syndrome. After stringent blood pressure management and the administration of sedatives and analgesics, symptoms were resolved in one case. However, in the other case, a cerebral hemorrhage occurred within 24 h, necessitating a craniotomy for hematoma evacuation and decompressive craniectomy. Fortunately, the patient survived, but was left with severe neurological dysfunction (mRS 4). Two patients experienced symptomatic distal embolism events, which resolved following aggressive medical treatment. A significant vascular dissection occurred in two cases following balloon dilation, which improved after stent implantation. In the failed recanalization group, the procedure was promptly terminated in two cases due to microguidewire-induced vessel perforation. Fortunately, no hemorrhagic complications ensued, and two patients did not exhibit any new symptoms upon awakening from general anesthesia. A case was terminated due to hemorrhage of the distal lenticulostriate artery area caused by hyperperfusion after vascular recanalization. The recanalized MCA was subsequently embolized with coils. Despite receiving intensive medical therapy postoperatively, the patient continued to experience severe neurological deficits, with a mRS score of 4 (Table 2).

**Table 2:** Periprocedural complications and follow-up outcomes.

**Variables**	**Total (*n* = 65)**	**Successful group (*n* = 50)**	**Failed group (*n* = 15)**
**Periprocedural complications**, ***n*** **(%)**			
Dissection	2 (3.1%)	2 (4.0%)	0 (0%)
Distal embolism	2 (3.1%)	2 (4.0%)	0 (0%)
Hyperperfusion syndrome	2 (3.1%)	2 (4.0%)	0 (0%)
ICH and/or SAH	2 (3.1%)	1 (2.0%)	1 (6.7%)
Vessel perforation	2 (3.1%)	0 (0%)	2 (13.3%)
Death	0 (0%)	0 (0%)	0 (0%)
**Follow-up outcomes**, ***n*** **(%)**			
Recurrent stroke/TIA	5 (7.7%)	2 (4.0%)	3 (20.0%)
Death	0 (0%)	0 (0%)	0(0%)
Significant ISR	/	1 (2.2%)	/
Minimal ISR	/	3 (6.5%)	/
Re-occlusion	/	1 (2.2%)	2 (100%)

ICH, intracranial hemorrhage; SAH, subarachnoid hemorrhage; TIA, transient ischemic attack; ISR, in-stent restenosis.

### Follow-up outcomes

Of the 65 patients, 63 (50 in the successful recanalization group and 13 in the failed recanalization group) underwent a 3-month clinical follow-up. The incidence of stroke within 3 months was 4.0% (2 out of 50) in the successful recanalization group. Among the 46 patients in the successful recanalization group who completed the 3-month imaging follow-up, 43 underwent a DSA examination, while 3 patients received a CTA examination at the outpatient clinic. One patient developed significant in-stent restenosis (ISR), while another experienced MCA re-occlusion. Additionally, minimal ISR (30%) was detected in three patients. In the failure recanalization group, only 2 patients who achieved mTICI grades of 2a post-operation completed the 3-month imaging examination, indicating that the vessels had re-occluded (Table 2).

### Subgroup analysis

As indicated in Table 1, concerning the clinical baseline characteristics of the patients, we observed no statistically significant differences between the successful and failure groups in terms of age, gender, vascular risk factors, ischemic events, baseline mRS, and NIHSS scores. Univariate analyses were conducted using logistic regression to ascertain associations with technical success (Table 1). The technical success rate was higher in patients with an occlusion duration of ≤ 3 months [odds ratio (OR): 4.095; 95% confidence interval (CI): 1.110–15.114; *P* = 0.034], an occlusion length of ≤ 10 mm [OR: 3.500; 95% CI: 1.057–11.586; *P* = 0.040], a tapered stump [OR: 3.882; 95% CI: 1.143–13.185; *P* = 0.030], a positive DVMTV sign [OR: 3.798; 95% CI: 1.061–13.587; *P* = 0.040], and a positive SDFA sign [OR: 5.107; 95% CI: 1.041–25.044; *P* = 0.044]. Subsequently, multivariate logistic binary regression analyses were conducted to determine the independent predictors of technically successful EVRT. The results, as presented in Table 3, indicated that an occlusion duration of ≤ 3 months (OR: 14.035; 95% CI: 1.528–128.890; *P* = 0.020), an occlusion segment length of ≤ 10 mm (OR: 22.555; 95% CI: 2.695–188.747; *P* = 0.004), the presence of a DVMTV sign (OR: 9.359; 95% CI: 1.176–74.472; *P* = 0.035), and the presence of a SDFA sign (OR: 9.143; 95% CI: 1.117–74.804; *P* = 0.039) were identified as independent positive predictive factors for technical success in EVRT of SNMCAO (Table 3).

**Table 3:** Logistic multivariate regression analysis of the predictors of technical success.

**Variables**	**Odds ratio**	**95% confidence interval**	***P* value**
Estimated occlusion duration (≤3 months)	14.035	1.528–128.890	0.020
Occlusion length (≤10mm)	22.555	2.695–188.747	0.004
Tapered-stump	2.448	0.475–12.614	0.284
DVMTV sign	9.359	1.176–74.472	0.035
SDAF sign	9.143	1.117–74.804	0.039

SDAF, slow distal antegrade flow; DVMTV, distal vascular main trunk visualization.

## Discussion

Based on the experience of treating acute MCA occlusion with EVRT, numerous scholars and institutions have found that EVRT is feasible for SNMCAO (7, 9, 10). However, the incidence of success in recanalization rates and periprocedural complications varies significantly, attributed to the differences in operation technique and case selection. This research was accomplished by two teams of senior neuro-interventional physicians, each with extensive clinical experience in the endovascular treatment of intracranial large artery stenosis or occlusion. Consequently, the impact of technical operational factors on the recanalization rate and the incidence of complications was minimized to the greatest extent possible. Thus, the appropriate selection of cases was the pivotal factor influencing the success rate of recanalization in this study. In this study, we discovered that a vascular occlusion time of ≤ 3 months, an occlusion segment length of ≤ 10 mm, a tapered stump, the presence of a DVMTV sign, and the presence of a SDFA sign were associated with successful EVRT. Through multivariate analysis, we further determined that all five of these factors were independent positive predictors for the technical success of EVRT in patients with SNMCAO. In particular, the DVMTV sign and SDFA sign were first proposed as predictors for EVRT of non-acute MCA occlusion.

The duration of occlusion is commonly considered a critical factor in determining the technical success rate of recanalization. Generally, as the duration of vascular occlusion increases, the degree of fibrosis and calcification in the occluded vessel segment becomes more pronounced, and the likelihood of recanalization failure also rises. The treatment experience with coronary artery CTO recanalization indicates that an occlusion time of ≤ 3 months is an independent predictive factor for successful recanalization (15, 16). Recent studies on the recanalization of non-acute MCA occlusion have also found that patients with occlusion times of ≤ 3 months have higher rates of successful recanalization (9, 10). In this study, the median time from initial radiological diagnosis to EVRT was 46 days (IQR 16–72 days). Univariate analysis revealed a certain correlation between occlusion time and successful recanalization. Multivariate analysis further confirmed that an occlusion time of ≤ 3 months was an independent predictive factor for successful recanalization (*P* = 0.020), which was largely consistent with the findings of some recent studies. However, considering the large confidence interval (95% CI: 1.528–128.890), our results may be unstable. On one hand, this study was retrospective, and there were certain limitations and difficulties in accurately estimating the occlusion time during the collection of clinical data. On the other hand, the sample size of this study was relatively small, with 80.0% of the patients having an occlusion time of ≤ 3 months. To enhance the stability of the results, future studies with larger sample sizes are needed to further substantiate and support the arguments.

Reports suggest that the length of the occluded segment is a crucial factor in determining the success of recanalization in coronary CTOs, with longer occlusions correlating with lower procedural success rates (14, 15). Numerous studies have shown that an occlusion length of >10 mm is an independent predictor of recanalization failure in coronary artery CTO (17, 18). During the univariate analysis, we identified a correlation between occlusion lengths of ≤ 10 mm and a high success rate of recanalization. Upon conducting further multivariate analysis, we established that an occlusion length of ≤ 10 mm independently predicted successful recanalization, comparable to the length of type I occlusion as per the new angiographic classification proposed by Gao et al. (7). They proposed a new angiographic classification for endovascular recanalization of SNMCAO, suggesting that type I occlusion (occlusion length ≤ 10 mm) had the highest recanalization success rate (95.5%) and the lowest complication rate (4.5%).

The morphology of the occlusion stump is also an important factor for endovascular recanalization. Most previous studies on the recanalization of CTOs in carotid and coronary arteries have consistently reported that a tapered stump of the occlusion segment facilitates the passage of the guidewire through the occluded segment, whereas a blunt or absent stump is an unfavorable factor for recanalization (16, 18–21). Katsuragawa et al. (22) have stated in their research that a tapered stump exhibits characteristics such as a small cavity recanalization zone, loose surrounding fibrous tissue, and a short occlusion segment. These features facilitate the smooth passage of the guidewire through the occlusion segment. However, in cases of blunt-shaped stumps or occlusions without a stump, there is no suitable landing point for the guidewire during exploration. This results in the guidewire being prone to bending, looping, and even entering the proximal branches of the occlusion. Currently, there is limited research on the impact of stump morphology on the success rate of endovascular recanalization treatment for SNMCAO. Chao et al. (23) reported that a tapered stump was an independent predictor for successful recanalization of non-acute occluded internal carotid arteries. Similar results were also reported in the study by Chen et al. (24). Gao et al. (7) found no significant difference in the success rate of recanalization between tapered and blunt stump occlusions in their study. This was attributed to the strong correlation between occlusion time and recanalization, which diminished the influence of stump morphology on the success of the procedure. In this study, univariate analysis demonstrated the advantage of a tapered stump for successful recanalization. Further multivariate analysis failed to confirm that a tapered stump was an independent predictor for successful recanalization. The reason leading to this result is consistent with those of the study by Gao et al. The occlusion time removed the influence of the occluded stump on the recanalization rate. The morphology of the occlusion stump is related to the duration of occlusion. Generally, the longer the occlusion time, the greater the likelihood of a blunt-type. However, due to the small sample size of this study, larger case numbers are still required in the future to verify this finding.

The DVMTV sign, a novel feature initially proposed in this article, refers to the imaging of the distal M1 main trunk vessel or the superior and inferior trunk vessels by the slow antegrade flow or retrograde collateral circulation filling of the contrast agent. The DVMTV sign can guide the direction of the micro-guidewire, preventing it from deviating significantly. When the micro-guidewire successfully passes through the occluded segment and enters the main trunk of the distal vessel, it indicates a very high probability that the micro-guidewire has traversed the true lumen within the occluded segment. In this study, among the patients who achieved reperfusion, 58% exhibited the DVMTV sign. Univariate analysis revealed that the DVMTV sign was significantly associated with the recanalization success rate (*P* = 0.040). Multivariate analysis further confirmed the DVMTV sign as an independent predictor (*P* = 0.035). However, additional cases will be needed in the future to determine whether there is a positive relationship between the DVMTV sign and the success rate of recanalization.

The SDAF sign, observed at the distal end of the occluded segment and also known as the thrombus outline sign, was initially described in the context of arterial thrombolysis studies (25, 26). It may result from the formation of microchannels within the thrombus in the occluded segment. This sign acts as an independent positive predictor of recanalization after arterial thrombolysis. It reflects not only the morphological characteristics of the thrombus but also indicates the local circulation around the occluded segment (27). During the late arterial phase of cerebral angiography, the SDFA sign is beneficial for guiding microguidewires to prevent deviation from the vessel's longitudinal axis and for ensuring smooth passage through the occluded segment, thereby reducing the risk of vascular perforation and dissection. In this study, there were 2 cases of vascular perforation events. In both instances, there were no signs of SDFA at the distal end of the occluded segment. Of all the patients in this study, a total of 24 exhibited signs of SDFA. Among these, 22 (91.7%) successfully achieved reperfusion, whereas 2 did not due to other factors. Univariate analysis revealed that the SDFA sign was closely related to the success of recanalization (*P* = 0.044). Upon conducting multivariate analysis, it was further determined that the SDFA sign served as an independent predictor (*P* = 0.039). As far as we know, this study was the first to verify the impact of the SDFA sign on the recanalization of SNMCAO. Future studies with a larger sample size are required to continue validating this result.

In addition to the aforementioned factors, the occlusion angle and the extent of calcification within the occluded segment are also significant determinants of the success rate for coronary artery CTO recanalization (15, 16, 28, 29). The greater the angulation of the occlusion and the more severe the calcification, the lower the success rate for endovascular recanalization becomes. Studies have shown that an occlusion angle of >90°, and severe occlusion calcification are independent predictors of recanalization failure in coronary artery and carotid CTO (29, 30). Thus, at the time of case inclusion, our two centers excluded cases with severe angulation and obvious calcification, thereby avoiding the influence of these two factors on the recanalization rate.

## Conclusions

In summary, an occlusion duration of ≤ 3 months, an occlusion length of ≤ 10 mm, the presence of the DVMTV sign, and the presence of the SDFA sign were considered independent positive predictive factors for the success of EVRT in patients with SNMCAO. These predictors can assist in patient selection and procedural planning, potentially enhancing the efficacy of endovascular treatment in this patient population. However, this study has its limitations. Firstly, it was a retrospective study with a small sample size, which may introduce potential selection bias and recall bias, affecting the final results. Secondly, the wide confidence intervals of the regression analysis in this study could result in an overestimation of the role of certain factors in successful recanalization. Consequently, prospective and multicenter studies with a larger sample size are required to further validate these findings in the future.

## Data Availability

The original contributions presented in the study are included in the article/supplementary material, further inquiries can be directed to the corresponding author.
